# Emotional Communication in Speech and Music: The Role of Melodic and Rhythmic Contrasts

**DOI:** 10.3389/fpsyg.2013.00184

**Published:** 2013-04-24

**Authors:** Lena Quinto, William Forde Thompson, Felicity Louise Keating

**Affiliations:** ^1^Department of Psychology, Macquarie UniversitySydney, NSW, Australia

**Keywords:** speech prosody, emotional communication, music cognition and perception, melodic variability index, normalized pairwise variability index

## Abstract

Many acoustic features convey emotion similarly in speech and music. Researchers have established that acoustic features such as pitch height, tempo, and intensity carry important emotional information in both domains. In this investigation, we examined the emotional significance of melodic and rhythmic contrasts between successive syllables or tones in speech and music, referred to as Melodic Interval Variability (MIV) and the normalized Pairwise Variability Index (nPVI). The spoken stimuli were 96 tokens expressing the emotions of irritation, fear, happiness, sadness, tenderness, or no emotion. The music stimuli were 96 phrases, played with or without performance expression and composed with the intention of communicating the same emotions. Results showed that nPVI, but not MIV, operates similarly in music and speech. Spoken stimuli, but not musical stimuli, were characterized by changes in MIV as a function of intended emotion. The results suggest that these measures may signal emotional intentions differently in speech and music.

Several commonalities exist in how emotion is expressed by speech prosody (tone of voice) and music (Sundberg, 1998). Both speakers and musicians convey emotion through cues such as timing, rate, intensity, intonation, and pitch. According to Scherer (1995), the reasons for this similarity may stem from the shared vocal constraints associated with speaking and singing. Based on a meta-analysis of 104 speech studies and 41 music studies, Juslin and Laukka (2003) concluded that similar changes in acoustic features occurred when conveying similar emotions in these domains. This observation led Juslin and Laukka to suggest that there is a “common code” for emotional expression in speech and music. Further evidence comes from studies that examined the emotional consequences of manipulating acoustic attributes. Ilie and Thompson (2006) reported that manipulations of pitch height, intensity, and rate (tempo) in speech and music yielded similar emotional ratings by listeners.

Comparisons of emotional features between the two domains have tended to focus on variables such as changes in intensity, duration, timbre, and pitch (Gabrielsson and Lindström, 2010; Juslin and Timmers, 2010). However, the full range of emotional cues, and their degree of overlap between speech prosody and music, has yet to be determined. The comparison of emotional attributes in music and speech has been challenging because direct analogs do not always exist. Speech and music may each have domain-specific cues to emotion, because they have different structural features and different functions. For example, in music pitches tend to be discrete and are typically organized hierarchically (Krumhansl, 1990). Pitches may also be specified by the composer and are not under the control of the performer. In speech, pitch movement tends to be continuous, not hierarchically organized and under the direct discretion of the speaker. Music is also characterized by regular cycles of stress, called meter. The deviations from expected timing contribute to the expressiveness of a musical performance (Palmer, 1997). In speech, rhythm is subtler, and debates exist as to how it is best quantified (see Patel, 2008). These issues represent a difficulty in comparing pitch and rhythm in affective speech and music. As a result, speech and music may each have shared and domain-specific cues to emotion, but only a relatively small number of the most obvious cues to emotion have been investigated.

Recently, two measures have been developed that allow an examination of the changing pitch and rhythmic properties of speech and music. The first, Melodic Interval Variability (MIV), is a measure of pitch variability. MIV takes into account differences in successive intervals (Patel, 2008). MIV is defined as the coefficient of variation (CV = standard deviation/mean) of absolute interval size for a sequence of tones. MIV yields a smaller value when interval changes are less variable, and a larger MIV value when interval changes are more diverse. This allows for comparisons between the variability of intervals in melodies independent of the average interval size.

The second measure, the normalized Pairwise Variability Index (nPVI), is a measure of rhythmic contrastiveness between successive durations (Low et al., 2000; Grabe and Low, 2002). It was developed to better understand the rhythmic differences found between languages, such as stress-timed versus syllable-timed languages (Low et al., 2000). Like MIV, a small nPVI indicates uniform durations between successive tones or syllables, whereas a greater nPVI indicates that successive durations are less uniform.^1^ The nPVI is an overall contrast value based on the length of successive syllables or tones.

These measures were developed independently, and variations of each measure exist. In the calculations documented by Patel et al. (2006), MIV is normalized with respect to the average interval between adjacent syllables or tones, and nPVI is normalized with respect to the average durations of adjacent syllables or tones.

MIV and nPVI measure distinct attributes – pitch and time – but have been examined together in the work of Patel and colleagues. Early anecdotal evidence suggested that a composer’s instrumental music was influenced by their nationality and language. However, because of difficulties in assessing the structural attributes of music, this hypothesis was difficult to test. Patel et al. (2006) used MIV and nPVI to compare the spoken language of a composer and the structural patterns found in their music. Patel et al. found that French speech has lower MIV and nPVI values than English speech. Similarly, music written by French composers has lower MIV and nPVI values than music written by English composers. These findings suggest that a composer’s language may influence the pitch and rhythmic properties of their music.

To date, there is no widely accepted account for why average measures of MIV and nPVI differ between languages, and there is little understanding of the degree to which these variables are perceivable. Patel (2008) offers a few reasons for the observed pattern of results. One reason for differences in MIV is that English speech may have more pitch levels than French speech – allowing for greater variability. Another reason is that composers may have internalized pitch and timing patterns in the speech of their culture and these patterns are reflected in their music. Research on rhythm and language suggests that nPVI differs between languages for a few reasons. One possibility could be due to varying amounts of vowel reduction by speakers, a second possibility could be differences in the proportion of vowels in a sentence, and a third possibility is that there may be differences in the variability of vowel types within a language (Patel et al., 2006). Additionally, there are currently no data on the degree to which people are sensitive to changes in MIV, but a study by Hannon (2009) indicates that participants can reliably classify sequences that vary in nPVI.

In summary, speech prosody and music are powerful channels of emotional communication. Previous research has found that MIV and nPVI are important attributes in both domains (e.g., Patel et al., 2006), yet the relevance of these attributes to emotional communication has never been examined. Our aims were (a) to determine whether these features carry emotional information in one or both domains and (b) to determine if they are associated with emotions in the same way in affective speech and music, or whether they operate differently in the two domains. First, we generated spoken and melodic stimuli conveying six emotional intentions. Next, stimuli were acoustically analyzed to assess differences in MIV and nPVI for each emotion and domain. We predicted that both measures would vary as a function of the intended emotion, but there are no clear grounds for making specific hypotheses. For example, it might be expected that high levels of MIV and nPVI would be associated with high-arousal emotions such as happiness and fear, because high values reflect greater pitch and durational contrasts. On the other hand, as there are no data to support such a hypothesis, the opposite could also be true. Melodies with consistently short durations (fast tempo, low nPVI) and consistently large pitch changes (low MIV) might also be expected in high-arousal emotions. Based on evidence that music and speech share a common emotional code, we predicted that these measures would carry similar emotional information in the two domains.

## Materials and Methods

### Spoken stimuli

Speakers were asked to emotionally express semantically neutral phrases such as “The boy and girl went to the store to fetch some milk for lunch.” Each sentence had 14 syllables and was expressed with the intention to communicate each of the six emotions of irritation, fear, happiness, sadness, tenderness, and neutral or no emotional expression. These emotions were selected because they have been identified as frequently used in previous studies (Juslin and Laukka, 2003) and involve a range of acoustic features.

#### Speakers

Six male and seven female speakers provided samples of emotional speech. Their average age was 23.65 years. All speakers were paid $15 for their participation.

#### Procedure

Speakers were asked to read a description of an affective scenario that was associated with one of the target emotions. We adopted this procedure to prepare speakers to verbally communicate the target emotion. Once they had read the scenario, they vocalized each of seven sentences while attempting to convey the intended emotion. This process was repeated for each emotion (irritation, fear, happiness, sadness, tenderness, or neutral). An experienced recording engineer provided feedback and coaching regarding the emotional expression of each sentence. The coaching did not involve suggestions for the use of cues to express emotion but rather encouragement to attempt additional renditions. Speakers were allowed to repeat each sentence until they, and the recording engineer were satisfied that the intended emotion was communicated.

#### Recording

Speakers were recorded in a professional recording studio at a sample rate and bit depth of 44.1 kHz/16 bit-mono. They spoke into a K2 condenser microphone (RØDE microphones) and were recorded with Cubase 4 (2008).

#### Pre-rating

Initially, 462 recordings were obtained (11 speakers × 6 emotions × 7 sentences). These recordings were then assessed in a pilot investigation involving 13 male and 22 female undergraduate students at Macquarie University (mean age = 21.49, SD = 4.75), with an average of 3.16 (SD = 4.00) years of musical experience. Participants heard a subset of the stimuli and made a forced-choice decision of the emotion they believed was conveyed. Their options were the six emotional intentions conveyed by the actors. Decoding accuracy was determined for every recording: The 16 most accurately decoded recordings were selected for each of the six emotions, resulting in 96 recordings balanced for speaker sex. This procedure was adopted to ensure that the intended emotions were expressed and to reduce the battery to a manageable size for analysis. The resultant battery of 96 spoken phrases (Macquarie Battery of Emotional Prosody, or MBEP) can be downloaded from the second author’s website at www.psy.mq.edu.au/me2. Table 1 summarizes some of the acoustic features associated with each intended emotion.

**Table 1:** **Means associated with the acoustic features of the Macquarie battery of emotional prosody (standard errors are shown in parentheses)**.

Acoustic feature (units of measurement)	Emotional portrayal					
	Anger/irritation	Fear	Happiness	Sadness	Tenderness	Neutral
F0 (Hz)	213.50 (13.23)	222.91 (24.70)	233.70 (13.78)	174.38 (18.29)	169.33 (18.86)	163.50 (14.95)
SD F0 (Hz)	41.35 (4.68)	27.86 (4.02)	58.89 (4.17)	21.67 (2.87)	33.12 (4.84)	28.02 (4.01)
Duration (s)	2.42 (0.08)	2.31 (0.08)	2.85 (0.12)	3.10 (0.13)	3.24 (0.15)	2.90 (0.11)
Intensity (dB)	73.76 (0.83)	74.80 (0.56)	73.99 (0.39)	68.76 (0.89)	68.76 (0.39)	71.66 (0.72)

### Musical stimuli

#### Musicians

Four violinists and four vocalists created the stimuli. Violin and voice were selected because both instruments allow musicians to use a wide range of performance features. All musicians were currently performing or had completed higher-level examinations for their instruments. The two vocalists who had not completed formal examinations had been actively singing for 17 and 20 years. On average, the musicians had 15 (SD = 3.89) years of formal training, with an average time of 21.15 (SD = 9.41) years performing.

#### Procedure

**Figure 1:**
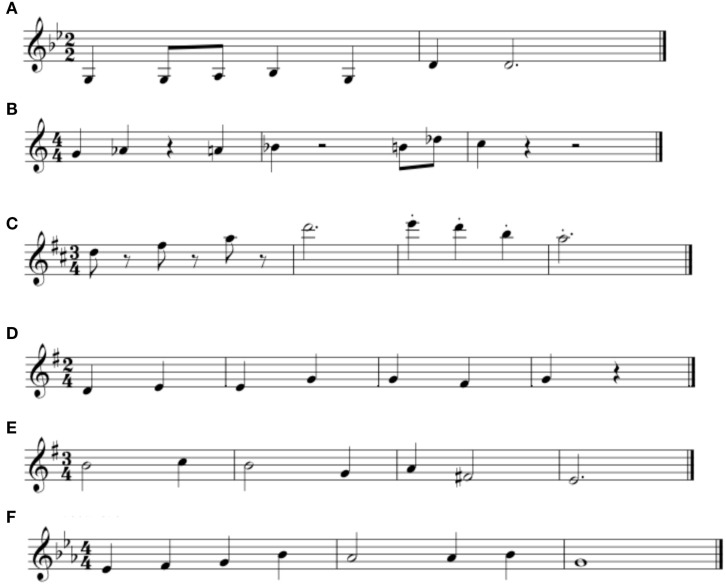
**Notated exemplars of the melodies expressing anger **(A)**, fear **(B)**, happiness **(C)**, neutral **(D)**, sadness **(E)**, and tenderness **(F)****.

We asked musicians to compose brief melodies with the intention of expressing the emotions of anger, fear, happiness, sadness, tenderness, and neutral. They were asked to compose melodies for their own instrument and to limit their compositions to a maximum of nine notes (range = 5–9 notes, average = 7.40 notes). Examples are illustrated in Figures 1A–F. In the *live condition*, musicians performed their own compositions in a manner that reinforced the emotion that was intended in each composition. In the *deadpan condition*, compositions were notated in MIDI format using Cubase. Deadpan compositions were recorded using timbres selected from a Roland super JV-1080 64 voice synthesizer with four expansion modules. Compositions produced by violinists were recorded using timbre 41 from the XPA preset bank (violin); compositions produced by vocalists were recorded using timbre 54 from the D (GM) preset bank (voice). The tempo of each melody in the deadpan condition was matched to the tempo as performed by the musician in the live condition. This procedure resulted in 96 stimuli (8 musicians × 6 emotions × 2 manners of performance). The stimuli in the live condition differed from the deadpan condition because performers had the ability to deviate from the notated pitch and rhythmic information. Two judges with at least 10 years of music training independently confirmed that the intended emotion was expressed in all cases. All musicians were paid $40 for their participation. In our study, pitch varied depending on emotional intention, whereas other properties of the sequence (whether verbal material or instrument timbre) were constant.

#### Recording of musical stimuli

Musicians were recorded in a quiet (testing) room at a sample rate and bit depth of 44.1 kHz/16 bit-mono. Performances were recorded using a K2 condenser microphone (RØDE microphones) and saved into Cubase 4 (2008). Table 2 summarizes some of the acoustic attributes associated with each intended emotion.

**Table 2:** **Means associated with the acoustic features of the musical stimuli (standard errors are shown in parentheses)**.

Acoustic feature (unit of measurement)	Emotional portrayal					
	Anger/irritation	Fear	Happiness	Sadness	Tenderness	Neutral
F0 (Hz)	318.07 (17.01)	426.32 (19.85)	443.91 (11.46)	414.56 (16.98)	445.98 (11.95)	386.60 (14.26)
SD F0 (Hz)	58.18 (5.58)	72.25 (7.96)	89.82 (9.80)	74.19 (5.31)	78.37 (7.53)	74.19 (5.32)
Duration (s)	4.91 (0.27)	6.82 (0.58)	3.41 (0.27)	6.78 (0.30)	6.56 (0.37)	4.51 (0.20)
Intensity (dB)	75.29 (1.02)	69.03 (1.56)	73.32 (1.61)	66.85 (1.78)	70.65 (1.22)	70.42 (1.61)

### Acoustic analysis

The first two authors parsed each sentence and musical phrase manually with text grids using Praat (Boersma and Weenink, 2010). Text grids marked the boundary of every syllable and note in each phrase. In both music and speech, large glides were not considered stable pitches and were ignored. MIV was computed by measuring the interval distance between two syllables or tones in semitones. For each syllable or tone, the mean frequency in hertz was calculated using Praat. The interval distance was then calculated in semitones using the formula: 12 × log2(Hz 1/Hz 2). Interval distances in semitones were then used to compute MIV by dividing the standard deviation in interval size by the mean interval size for each sentence or musical phrase. The nPVI was computed by measuring the duration of each syllable or tone from its onset to the onset of the next syllable or tone. Periods of silence were included in the calculation of nPVI but not for MIV.

A short comparison of some acoustic features measured in both sets of stimuli (mean fundamental frequency, the standard deviation in fundamental frequency, duration, and intensity) revealed that there were similar changes in both domains depending on emotion. However, some exceptions did occur. For example, in music, anger was associated with a low pitch, whereas in speech, irritation (or mild anger) was associated with a high pitch. There are two potential reasons for the observed differences. The first is that any acoustic feature is associated probabilistically with emotional expression. Thus, the use of any one feature in the expression of an emotion may change depending on the rendition or portrayal. A second reason may be the semantic labels associated with the emotions. Note that five of the emotions in the two stimulus sets were the same. However, whereas the speech prosody stimulus set included the negative emotion of irritation, the music stimulus set included the (more intensely negative) emotion of anger. In other words, one emotion category differed in the intensity of the emotion. This difference in semantic labels arose inadvertently because the two stimulus sets were developed independently.

There were some features that were not common to both sets of stimuli. For example, in the spoken stimuli there was a downward pitch trend for phrases intended to communicate irritation and an upward pitch trend for phrases intended to communicate happiness. This was referred to as slope and was an average measure of the pitch movement. The spoken stimuli also differed in the number of pitch direction changes that occurred. Phrases expressing happiness had a greater number of pitch changes than those expressing irritation.

In the music stimuli, pieces intended to have a negative valence (i.e., anger, sadness, and fear) were more strongly correlated with a minor mode than pieces intended to have a positive valence (i.e., happiness, tenderness). There was also a trend whereby the average interval size was larger for pieces expressing happiness than for pieces expressing sadness. Additional details can be found in Thompson et al. (2012) and Quinto et al. (in press).

## Results

### Spoken stimuli

Separate linear mixed effects models were conducted for the spoken and musical stimuli, and for the two dependent variables. The 96 recordings were the observations in each analysis. A linear mixed effects analysis was selected because the stimuli did not reflect independent observations and because the spoken stimuli did not have equal numbers or equal speakers of each sentence. It was important to account for the effects of using the same speaker (or musician) and sentence repeatedly. Stimuli that used the same speaker or the same sentence might be expected to be more similar (correlated) than stimuli that differ with respect to these variables. For the spoken stimuli, the variable of sentence and speaker were entered as random effects (intercepts). For the musical stimuli, the variable of performer was entered as a random effect (intercept).

For the spoken stimuli, the linear mixed effects analysis with MIV as the dependent variable and emotion as a fixed effect revealed a significant main effect of emotion, *F*(5, 82.86) = 4.27, *p* = 0.002. Means and standard deviations are shown in Table 3. Pairwise tests with Bonferroni correction showed that expressions of happiness had a lower MIV value than expressions of sadness, *t*(82.86) = 3.84, *p* < 0.001; and tenderness, *t*(82.86) = 3.47, *p* < 0.001. No other significant differences emerged. The results suggest that portrayals of happiness in speech are associated with relatively low variability in successive interval size, whereas portrayals of sadness and tenderness are associated with higher variability in interval size. The covariance parameter indicated that the sentence standard deviation was (range of the intercept) 2.45 (Wald *Z* = 0.28, *p* = 0.78). This suggests that the variation between sentences was small. The addition of speaker as a random effect showed that the covariance parameter was redundant suggesting that there was not enough variance or that the variances in speakers were highly correlated.

**Table 3:** **The means for each of the six emotions and acoustic features (standard errors are in parentheses)**.

Acoustic feature	Emotional portrayal					
	Anger/irritation	Fear	Happiness	Sadness	Tenderness	Neutral
MIV speech	81.23 (3.88)	74.85 (4.07)	63.93 (2.98)	91.08 (6.17)	88.51 (4.54)	85.21 (7.13)
MIV music	74.46 (5.96)	71.32 (9.06)	74.52 (7.63)	67.58 (3.47)	63.57 (5.35)	66.38 (6.63)
Interval size (speech)	2.42 (0.12)	1.64 (0.15)	4.46 (0.29)	2.37 (0.46)	2.98 (0.32)	2.06 (0.52)
Interval size (music)	3.23 (0.32)	2.53 (0.39)	3.22 (0.34)	2.29 (0.11)	2.82 (0.24)	2.45 (0.22)
nPVI speech	58.81 (4.68)	54.53 (4.45)	54.38 (4.68)	57.86 (4.64)	61.74 (4.66)	52.92 (4.64)
nPVI music	56.22 (6.61)	46.39 (6.28)	52.01 (3.72)	56.59 (3.91)	46.44 (4.56)	31.19 (4.59)

*Note that the means are unweighted for all variables except nPVI in the spoken condition*.

*Unit for interval size is semitones*.

The average interval size was also assessed to demonstrate the independence of information provided by the variables of MIV and average interval size. A second linear mixed effects model with the average interval size as the dependent variable, and speaker and sentence as random variables also revealed a significant main effect of emotion, *F*(5, 86.92) = 15.72, *p* < 0.001. Pairwise tests revealed that happiness had a greater average interval size than all other emotions, *t*’s(86.92) > 3.12, *p* < 0.001. This finding demonstrates that MIV and average interval size provide different types of information in emotional speech. Specifically, while happiness might be associated with low variability in interval size, the types of intervals that are associated with the expression of happiness tend to be larger as compared to other emotions. Similarly, sadness and tenderness were associated with smaller to intermediate interval sizes yet relatively higher MIV values were associated with these emotions. The covariance parameter indicated that the sentence standard deviation was 0.28, (Wald *Z* = 0.81, *p* = 0.42).

A linear mixed effects model with nPVI as the dependent variable, emotion as the fixed effect and sentence and speakers as random effects revealed a significant effect of emotion, *F*(5, 81.37) = 2.88, *p* = 0.02. This effect arose from neutral or “no emotion” expressions having a lower nPVI value than the emotional expressions. Tests of simple effects with Bonferroni correction showed that “no emotion” expressions have a significantly lower nPVI than tenderness, *t*(81.37) = 3.19, *p* = 0.05. The means for the spoken nPVI in Table 3 reflect estimated marginal means that take into account the effects of sentence. The covariance parameter indicated that the speaker standard deviation was 3.86 (Wald *Z* = 1.48, *p* = 0.14) and that the sentence standard deviation was (intercept) 10.80 (Wald *Z* = 1.67, *p* = 0.09). The relatively large standard deviation suggests that there was considerable variance between the sentences.

### Musical stimuli

For the musical stimuli, we conducted a mixed linear effects model with MIV as the dependent variable, mode of presentation (live versus deadpan), and emotion as the independent variables. Performer was treated as a random factor. There was no effect of mode of presentation, *F*(1, 77) = 0.14, *p* = 0.71, nor was there an effect of emotion, *F*(5, 77) = 0.54, *p* = 0.70. The interaction between emotion and mode of presentation also was not significant, *F*(5, 77) = 0.14, *p* = 0.98. This finding suggests that MIV did not distinguish emotional portrayals in music. The covariance parameter indicated that the standard deviation for performer was 13.80 (Wald *Z* = 1.51, *p* = 0.13).

The linear mixed effects model with nPVI as the dependent variable revealed a significant main effect of mode of presentation, *F*(1, 77) = 9.39, *p* = 0.003. The nPVI was significantly higher in the live condition (*M* = 54.03, SD = 22.48) than in the deadpan condition (*M* = 42.26, SD = 19.03). This finding demonstrates that performers enhanced the durational contrasts between successive tones when performing their compositions as compared to the deadpan renditions. There was also a significant main effect of emotion, *F*(5, 77) = 4.03, *p* = 0.003. As shown in Table 3, nPVI was significantly lower for neutral expressions than for melodies conveying anger, *t*(77) = 3.55, *p* < 0.001; sadness, *t*(77) = 3.60, *p* < 0.001; and (marginally for) happiness, *t*(77) = 2.15, *p* = 0.056. The interaction between emotion and mode of presentation was not significant, *F*(5, 77) = 0.14, *p* = 0.98. The covariance parameter indicated that the standard deviation for performer was 6.62 (Wald *Z* = 1.11, *p* = 0.27).

## Discussion

The results of this investigation confirm that both MIV and nPVI can reflect emotional intentions. MIV varied as a function of emotional intentions in speech but not in music, whereas nPVI differentiated emotional from non-emotional portrayals in both domains. While similarities have been documented in the expression of emotion in speech and music (Scherer, 1995; Juslin and Laukka, 2003; Bowling et al., 2010; Curtis and Bharucha, 2010) and emotional experiences (Ilie and Thompson, 2011; Coutinho and Dibben, 2012), the current finding represents differences in the use of pitch contrasts as emotional information in the two domains. Differences between music and speech in the cues used to communicate emotions are hardly surprising: music contains features that have no clear analog in speech, such as harmony and the tonal hierarchy. However, it was observed that there were similarities in the use of nPVI to differentiate emotional from non-emotional sentences and musical phrases.

In speech, MIV values were lower for portrayals of happiness than for portrayals of sadness, tenderness, and neutral. This means that changes in interval size were more uniform for happiness than the other emotions. Yet portrayals of happiness had the greatest average interval size as compared to other emotional portrayals (see also Banse and Scherer, 1996). From a physiological perspective, greater pitch variability may reflect a higher arousal level but the consistency of interval changes may reflect the speaker’s ability to control the regularity of the pitch. Since acoustic features only probabilistically contribute to the expression of a given emotion, and do not always behave consistently, this finding contributes another acoustic feature that could be used to differentiate emotions in speech.

An analysis of musical stimuli revealed no significant effect of emotion on MIV. Thus, whereas previous studies have found that pitch-based cues such as pitch height, pitch range, pitch variability, and modality contribute to emotional communication (Ilie and Thompson, 2006; Gabrielsson and Lindström, 2010; Quinto et al., in press), MIV does not appear to be involved in the communication of emotion in music. Nonetheless, recent investigations suggest that features considered to be music-specific, including interval size and mode, may actually play a role in emotional speech (Bowling et al., 2010; Curtis and Bharucha, 2010). Specifically, excited and happy speech have been found to contain a higher proportion of major intervals (happy sounding) than minor intervals (sadder sounding) and sad speech has been found to contain a greater proportion of minor intervals than major intervals. One potential reason for the null result is that conventional associations between pitch structure and emotion, such as modality, may guide the creation of emotional music. Hence, the extent to which other pitch cues, such as MIV could vary may be restricted.

For speech, nPVI was significantly greater for emotional utterances than for neutral or non-emotional utterances. One interpretation of this finding is that durational contrasts function to attract and maintain attention, enhancing sensitivity to emotional messages. Changing-state sounds, including changes in duration as measured by nPVI, are known to capture attention (Jones and Macken, 1993). By capturing attention, nPVI may fulfill a primary goal of emotional communication, increasing the capacity of speakers to influence the perceptions and behaviors of others (Bachorowski and Owren, 2003).

As in the speech stimuli, nPVI values in music were significantly lower in deadpan melodies than live recordings, indicating that the use of performance expression involved enhancing rhythmic contrasts between tones relative to strict notation. This increase in nPVI for performed melodies occurred for all intended emotion categories: there was no significant interaction between emotion and mode of presentation. There was also a significant main effect of emotion on nPVI. This effect was driven primarily by a comparatively large difference between mean nPVI values for neutral and emotional music, regardless of the mode of presentation. Among the five non-neutral (emotional) portrayals, differences between the nPVI values were relatively small and not statistically reliable in *post hoc* analyses. Taken together, nPVI was primarily effective in distinguishing (a) performed from deadpan music, and (b) emotional from non-emotional music.

These findings demonstrate that the processes of both composition and performance independently contribute to changes in rhythmic contrasts. Performance expression introduces these rhythmic contrasts regardless of emotional intention. This finding is compatible with the “duration contrast” rule in the KTH rule system for musical performance, proposed by Sundberg and colleagues (Sundberg et al., 1982; Thompson et al., 1989; Friberg et al., 2006). This finding also extends research reported by Gabrielsson and Juslin (1996), who observed that emotional performances tend to be characterized by exaggerated timing deviations and durational contrasts. Our results suggest that nPVI provides an effective quantification of this expressive phenomenon.

A novel finding of this investigation is that emotional music, whether performed or deadpan, is characterized by increased durational contrasts as measured by nPVI. Compared to music that was composed to be emotionally neutral, music composed to be emotional contained increased durational contrasts.

The current data confirm that MIV and nPVI are associated with emotional communication in music and speech, but the extent to which these attributes are actually used by listeners for decoding has yet to be determined. Evidence does suggest that listeners are able to perceive differences in nPVI (Hannon, 2009) and unpublished work in our lab suggests that participants can differentiate levels of MIV. However, the extent to which these attributes aid listeners in decoding emotion is uncertain.

A direction for future research might be to assess the role of MIV and nPVI when other cues are restricted. For example, MIV may play a greater role in emotional expression for atonal music, for which influences by modality and the tonal hierarchy are absent. It is also unclear whether the results for our stimuli can be generalized to speech and music produced naturally (Scherer et al., 2003). Finally, given the cross-cultural findings of Patel et al. (2006), it seems possible that the emotional connotations of MIV are not only domain-specific but may operate differently in different languages. For example, it is possible that when emotional English is compared to emotional French, then the use of MIV as an emotional cue may be observed. While emotional decoding across cultures is relatively good, individuals within a culture are still better able to identify emotion than outsiders. These cultural differences in emotional communication are referred to as “pull-effects” (Scherer et al., 2003) and can account for the experience of non-native speakers of a language misunderstanding the emotional intentions of native speakers (Wierzbicka, 1999; Mesquita, 2003).

We note that there are some differences between our spoken and musical stimuli that could have influenced the results. One difference between the two stimulus sets concerns the semantic labels used for one of the emotions (anger versus irritation). A second difference concerns the manner in which the stimuli were selected. It may be useful in future research to examine spoken and musical stimuli that have been developed using similar selection criteria and have matching semantic labels.

It could be argued that differences in the attributes involved in musical and spoken stimuli may account for some of the effects observed. Specifically, because spoken material contained words and the musical material involved instrument timbres, these properties may have exerted an influence on the pitch (MIV) and timing (nPVI) of the stimuli. Such influences could occur if, for example, sentences varied in the number of words that are naturally spoken with different melodic intonation (regardless of any emotional intention), and musicians composed melodies with different degrees of durational contrast depending on the instrument that they played. However, our analyses took into account these possible effects.

To conclude, nPVI reflects a “common code” of emotional expression in music and speech but MIV does not. Based on observations by Patel et al. (2006) and Juslin and Laukka (2003) it was expected that both these measures would change similarly in both speech and music. MIV may be a useful predictor of emotional speech whereas the nPVI may help to differentiate emotional from non-emotional music and speech. The use of universal cues (e.g., loudness, pitch, tempo) may allow individuals to decode unfamiliar emotional speech (Elfenbein and Ambady, 2002) and music (Balkwill and Thompson, 1999; Fritz et al., 2009). However, it is possible that attributes of music and speech have domain-specific constraints, which may mean that only some cues are effective. The task of differentiating universal, domain-specific, and culture-specific cues to emotion is an exciting challenge for future research.

## Conflict of Interest Statement

The authors declare that the research was conducted in the absence of any commercial or financial relationships that could be construed as a potential conflict of interest.

## Footnotes

^1^The following equation shows how the nPVI is calculated:

$$\text{nPVI}=\frac{100}{m-1}\times \overset{m-1}{\underset{k=1}{\sum }}\left|\frac{d_{k}-d_{k+1}}{\frac{d_{k}+d_{k+1}}{2}}\right|,$$

*M* is the number of durational elements in a sequence and *d_k_* is the duration of the *k*th element. Individual contrast values between each syllable or tone are computed, summed and averaged to yield the nPVI for a entire sentence.
